# Phenotypic Differences Between the Epidemic Strains of Vesicular Stomatitis Virus Serotype Indiana 98COE and IN0919WYB2 Using an In-Vivo Pig (*Sus scrofa*) Model

**DOI:** 10.3390/v16121915

**Published:** 2024-12-13

**Authors:** Kate Hole, Patrycja Sroga, Michelle Nebroski, Katherine Handel, Oliver Lung, Edward Spinard, Selene Zarate, Charles Nfon, Luis L. Rodriguez, Shawn Babiuk, Chad Mire, Lauro Velazquez-Salinas

**Affiliations:** 1 National Center for Foreign Animal Disease (NCFAD), Canadian Food Inspection Agency, Winnipeg MB R3E 3M4, Canada; kate.hole@inspection.gc.ca (K.H.); patrycja.sroga@inspection.gc.ca (P.S.); michelle.nebroski@inspection.gc.ca (M.N.); katherine.handel@inspection.gc.ca (K.H.); oliver.lung@inspection.gc.ca (O.L.); charles.nfon@canada.ca (C.N.); shawn.babiuk@inspection.gc.ca (S.B.); 2 Department of Biological Sciences, University of Manitoba, Winnipeg, MB R3T 2M8, Canada; 3 Foreign Animal Disease Research Unit, USDA/ARS Plum Island Animal Disease Center, P.O. Box 848, Greenport, NY 11944, USA; edward.spinard@usda.gov; 4 Posgrado en Ciencias Genómicas, Universidad Autónoma de la Ciudad de Mexico, Ciudad de Mexico 03100, Mexico; selenezarate@uacm.edu.mx; 5 National Bio- and Agro-Defense Facility, Agricultural Research Services, United States Department of Agriculture, Manhattan, KS 66506, USA; luis.rodriguez@usda.gov

**Keywords:** virulence, vesicular stomatitis virus, evolution, pathogenesis, epidemics, transmissibility

## Abstract

During the past 25 years, vesicular stomatitis virus (VSV) has produced multiple outbreaks in the US, resulting in the emergence of different viral lineages. Currently, very little is known about the pathogenesis of many of these lineages, thus limiting our understanding of the potential biological factors favoring each lineage in these outbreaks. In this study, we aimed to determine the potential phenotypic differences between two VSV Indiana (VSIV) serotype epidemic strains using a pig model. These strains are representative of the epidemic lineages that affected the US between 1997 and 1998 (IN98COE) and between 2019 and 2020 (IN0919WYB2), the latter responsible for one of the most extensive outbreaks in the US. Our initial genome analysis revealed the existence of 121 distinct mutations between both strains, including the presence of a 14-nucleotide insertion in the intergenic region between the G and L genes observed in IN0919WYB2. The levels of viral RNA in clinical samples between pigs infected with IN98COE or IN0919WYB2 were compared. Overall, higher and prolonged expression of viral RNA in pigs infected with IN98COE was observed. However, clinically, IN0919WYB2 was slightly more virulent than IN98COE, as well as more efficient at producing infection through contact transmission. Additionally, infectious virus was recovered from more samples when the pigs were infected with IN0919WYB2, as revealed by virus isolation in cell culture, indicating the increased ability of this virus to replicate in pigs. Sequence analyses conducted from isolates recovered from both experimental groups showed that IN0919WYB2 produced more variability during the infection, denoting the potential of this strain to evolve rapidly after a single infection–contact transmission event in pigs. Collectively, the results showed that epidemic strains of VSIV may represent disparate phenotypes in terms of virulence/transmissibility for livestock, a situation that may impact the intensity of an epidemic outbreak. This study also highlights the relevance of pathogenesis studies in pigs to characterize phenotypic differences in VSV strains affecting livestock in the field.

## 1. Introduction

Vesicular stomatitis (VS) is a viral disease that annually affects livestock in the Americas [1] and can cause sporadic large outbreaks in the US [2,3]. VS is an arboviral disease caused by the vesicular stomatitis virus (VSV), a negative sense RNA virus belonging to the *Rhabdoviridae* family, genus *Vesiculovirus* [4]. The genome of VSV contains five genes encoding five proteins: nucleoprotein (N), phosphoprotein (P), matrix (M), glycoprotein (G), and the large RNA-dependent polymerase (L) [5]. Additionally, diverse nonstructural proteins are encoded by alternative reading frames in the P (proteins C’ and C) [6] and M (M2 and M3 proteins) [7] genes. Two main serotypes have been described: VSV New Jersey virus (VSNJV) and VSV Indiana virus (VSIV) [1]. Pathogenesis studies conducted in pigs have concluded that the VSNJV strains are generally more virulent than VSIV [8]. However, a recent pathogenesis study showed that some VSIV strains could be as virulent as VSNJV [9], indicating that more pathogenesis studies are needed to gain insights into the phenotypic differences among VSV strains.

VSV is maintained in well-established endemic settings in Mexico and multiple countries in Central America, where clinical cases are reported annually in livestock [1]. Outbreaks of VSNJV in the US have been documented during 1995–1997, 2004–2006, and 2012–2015 [3]. VSIV has been associated with outbreak events occurring in 1997–1998 [10] and 2019–2020 [2]. In all cases, these outbreaks have been related to single monophyletic lineages, which display a high level of genome identity with viral strains that circulate in the endemic zones of Mexico [3,4].

Very little is known about the molecular factors associated with the emergence of epidemic lineages in the US and the potentially diverse viral phenotypes associated with these viruses. A previous pathogenesis study in pigs suggested that an epidemic strain of VSNJV observed in the US (Strain NJ0612NME6, lineage 1.1) might represent a more virulent phenotype than its endemic relative (Strain NJ0806VCB, lineage 1.2) [11]. This virulent phenotype was associated with an increased number of vesicular lesions produced during the acute stage of the disease, a condition linked to the potential ability of the epidemic strain to better evade the host’s innate immune response [11].

Based on the current evidence, it can be hypothesized that during epidemics, the maintenance and spread of epidemic lineages is achieved by infections in both livestock and insect vectors [12]. Within affected farms, VSV can be maintained in herd animals by direct contact with infected animals or fomites [12]. In the absence of viremia, the role of livestock as amplifier hosts has been questioned, suggesting that these species are the end hosts in the natural cycle of VSV [1]. However, alternative mechanisms have been experimentally proposed, pointing to livestock as non-conventional amplifier hosts [13,14,15]. These mechanisms imply that livestock are a virus source during vector feeding from active vesicular lesions or infected tissues without lesions [15]. This observation suggests that VSV strains showing increased levels of virulence in livestock may have potential adaptive advantages, allowing them to survive and thrive in epidemic conditions and thus possibly impacting the intensity of the outbreak events. This advantage might be associated with increased levels of replication in the vertebrate host, providing an optimal condition to produce new viral variants during the infection, a situation poorly explored until now.

**Figure 1:**
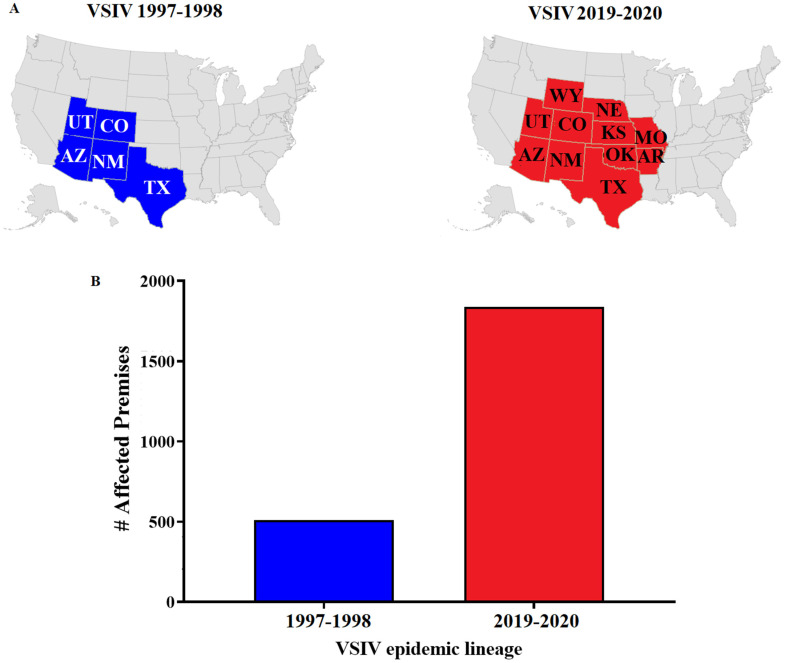
Summary of the epidemiological dynamics of two distinct epidemic VSIV lineages affecting the US during the 1997–1998 and 2019–2020 outbreaks. (**A**) Geographical distribution of the VSIV 1997–1998 and VSIV 2019–2020 lineages associated with the strains 98COE and IN0919WYB2, respectively. States are abbreviated as Arizona (AZ), Arkansas (AR), Colorado (CO), Kansas (KS), Missouri (MO), Nebraska (NE), New Mexico (NM), Oklahoma (OK), Texas (TX), Utah (UT), and Wyoming (WY). (**B**) Differences between the number of affected premises during the 1997–1998 and 2019–2020 VSIV outbreaks in the US.

The emergence and evolution of an epidemic VSIV lineage in the US was recently observed during the 2019–2020 outbreak [4]. After more than 20 years of absence, this lineage emerged in the US and produced one of the largest outbreaks in the US (Figure 1) [2]. This epidemic lineage was highly related to VSIV strains from endemic zones in Mexico. Throughout the outbreak, it diverged into four distinct phylogenetic groups [4]. A unique feature of this lineage is the presence of a 14–22 nucleotide insertion in the intergenic region between the G and L genes, a characteristic that was observed also in the ancestral endemic relatives of this lineage [4]. Based on the high number of clinical cases reported during the outbreak [2], it is possible to hypothesize that the epidemic VSIV 2019–2020 lineage may represent a virulent phenotype of VSIV in livestock.

This study aimed to determine the phenotypic characteristics of the VSV strain IN0919WYB2, a virus representative of the epidemic VSIV lineage from 2019–2020. For this purpose, a pathogenesis study was conducted using a well-established pig model [11,16,17], as pigs are a natural host of VSV. The VSIV strain 98COE, a virus related to the epidemic US lineage from the 1997–1998 outbreak, was used as a control. Epidemiologically, this lineage produced a considerably lower number of affected premises than lineage 2019–2020 (Figure 1). In this context, based on the epidemiological dynamics of the 2019–2020 VSIV outbreak, we hypothesized that the viral strain IN0919WYB2 represents a more virulent phenotype in pigs than the strain 98COE.

## 2. Materials and Methods

### 2.1. Viruses and Cells

The field VSIV strains 98COE (horse; CO, USA) and IN0919WYB2 (bovine; Garland, WY, USA) were used for this study. Both strains were collected from vesicular lesions produced by natural infections during the outbreaks of VSIV in the USA in 1998 and 2019, respectively. Viral stocks were produced in Vero-76 cells passage 38. Stocks were stored at −70 °C until needed.

### 2.2. Phylogenetic and Evolutionary Analyses

Phylogenetic analyses presented in this study used a maximum likelihood method based on a general time reversible model, with 100 bootstrap replicas as statistical support for the tree topology. Analyses were conducted using the MEGA software version 10.2.5 [18]. Additionally, nonsynonymous and synonymous pairwise distance analyses were performed using Sequence Distances in the SSE software version 1.2 [19].

### 2.3. Animal Experiments

**Figure 2:**
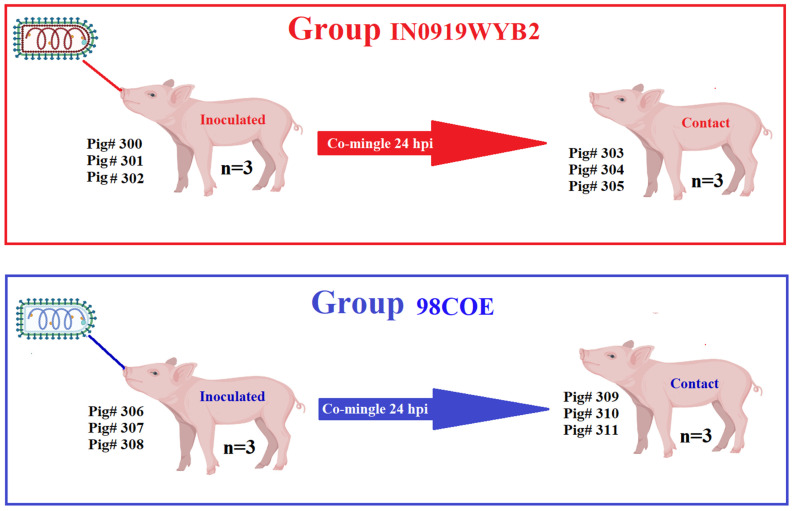
General overview of the experiment design. Two groups of pigs were used to evaluate the pathogenesis of the strains 98COE and IN0919WYB2, representative of the epidemic lineages responsible for outbreaks of VSIV in the US during 1997–1998 and 2019–2020. Each experimental group comprised six pigs, with three of the pigs in each group being inoculated in the snout. In comparison, the remaining three pigs evaluated the ability of each strain to be transmitted by direct contact. Contact pigs in each room co-mingled with inoculated pigs after 24 h post-inoculation (hpi). Pig# reflects diverse pigs’ roles during the experiment (inoculated or contact). This figure was created using BioRender.com under the agreement number YH27EC6GAL.

Pathogenesis experiments in pigs were conducted as previously described [11,16,17]. The main goal was to compare the virulence levels of 98COE and IN0919WYB2 by evaluating the capability of these viruses to infect pigs both by direct inoculation (scarification of the snout) and infection by direct contact between infected and naïve animals (Figure 2). Signs of virulence were evaluated using clinical parameters including the presence of fever, development of vesicular lesions, as well as the shedding of infectious particles recovered from nasal, oral, and rectal swabs. For vesicular lesions, the evaluation was based on the ability of these viruses to produce secondary lesions in inoculated animals. The number of animals used in this study was chosen based on previous studies evaluating the pathogenesis of VSNJV and VSIV in pigs [11,16,17].

The studies were conducted at the National Centre for Foreign Animal Disease (NCFAD), a biosafety level 3 agricultural (BSL-3Ag) facility that is part of the Canadian Food Inspection Agency. Two duplicate in vivo experiments were conducted, one with each representative strain. In each experiment, six landrace/duroc pigs (~7 weeks old and 13–15 kg in weight) were housed together in the same animal room. After one week of acclimation, on the inoculation day, three of the pigs (contact group) were moved into a different room to avoid contact with the inoculated pigs for 24 h. The three remaining pigs (inoculated group) in the room were sedated using 0.3 mL of Stresnil (40 mg/mL Azaperone, Elanco Canada Ltd., Mississauga, ON, Canada) administered intramuscularly in the left hamstring. Afterward, pigs were anesthetized with isoflurane in oxygen. Inoculation was conducted by scarification of the snout via microneedling with a 20 G needle up to the bevel of the needle. The snout was pricked 20 times prior and after the addition of 10^7^ TCID_50_ of virus (in 50 μL of Alpha Minimal Essential Medium ((AMEM), Wisent Inc., Saint-Jean-Baptiste, QC, Canada) on the scarified area. The inoculum was allowed to soak for 3 min. The remaining inoculum was returned to the lab for back titration. After 24 h, contact pigs were returned to the room to co-mingle with the inoculated group for the duration of the experiment (21 days) (Figure 2).

### 2.4. Clinical Evaluation and Sample Collection

Animals were clinically evaluated and sampled daily from day −1 (basal collection) through day 10 and again at 16 and 21 days post-infection (dpi). Clinical evaluation consisted of measuring rectal temperature before sample collection and identifying vesicular lesions. The severity and dissemination of vesicular lesions were assessed using a clinical score system previously described [11,16,17]. Lesions on the feet at each digit contributed two points. An additional point was counted for vesicular lesions observed in the snout of inoculated pigs, and two points were counted for snout lesions in the case of contact animals. Additionally, two points were added for lesions in the carpal/tarsal skin, lips, and oral cavity.

The sampling strategy included collecting blood samples obtained from the jugular vein into EDTA-containing tubes or without anticoagulant to obtain serum. Oropharyngeal swabs were collected by targeting the tonsils of the soft palate using a large cotton swab. Nasal swabs were collected by swiping a small cotton swab within the external nares. Additionally, rectal swabs were collected from each pig using a small cotton swab. Swabs were broken off in universal transport media.

Based on animal welfare protocols at CSCHAH, blood, oropharyngeal, and rectal swabs were collected every other day (−1, 2, 4, 6, 8, 10, 16, and 21 dpi). For this purpose, all pigs were sedated and anesthetized as described in the animal experiment subsection. Nasal swabs were collected daily with no requirement for sedation.

### 2.5. Postmortem Sample Collection

At 21 dpi after being sedated and anesthetized for sample collection, all six pigs were euthanized with 20 mL of 240 mg/mL sodium pentobarbital (Euthanyl, Bimeda MTC, Cambridge, ON, Canada) administered intravenously. Necropsies were conducted on all animals, and the following tissues were collected for analysis: tonsil of the soft palate, submandibular lymph node, popliteal lymph node, snout skin, neck skin, liver, spleen, anterior tongue epithelium, gastrohepatic lymph node, parotid lymph node, nasopharyngeal tonsil, and prescapular lymph node. Tissues were placed in individual tubes and frozen at −70 °C until further processing.

### 2.6. Viral RNA Detection

Viral RNA was extracted using the MagMax™-96 viral RNA isolation kit (AM1836, Applied Biosystems/Thermo Fisher Scientific, Ottawa, ON, Canada) and a KingFisher^TM^ Automated Purification Instrument (Thermo Fisher Scientific) following the manufacturer’s instructions for all clinical samples collected during this study. Specific detection and quantification of 98COE and IN0919WYB2 strains was carried out using a previously published multiplex real-time reverse-transcription polymerase chain reaction assay (RT-qPCR) protocol [20]. The quality of the RNA extractions was evaluated by real-time reverse-transcription polymerase chain reaction assay (RT-qPCR) using the β-actin gene as a target for the reaction by modifying a previously published protocol [21]. Briefly, the primers and probe remained static as per the publication. The master mix and cycling conditions were modified to mimic the VSV RT-qPCR conditions. TaqMan™ Fast Virus 1-Step Master Mix kit (REF#4444434, Applied Biosystems, Thermo Fisher Scientific) was used in a final volume of 25 μL. Amplification conditions for this kit were as follows: For reverse transcription, one cycle of 5 min at 50 °C followed by 95 °C for 20 s, and then, 45 cycles of 95 °C for 15 s and 60 °C for 45 s (data collection). All assays were run on the QuantStudio 7 Pro (Applied Biosystems, Thermo Fisher Scientific).

### 2.7. Detection of Viral Antigen

The detection of viral antigen from clinical samples was conducted using a double antigen-detection sandwich ELISA (DAS ELISA) following a previously described protocol [22]. OD values > 0.1 were considered positive for antigen.

### 2.8. Virus Isolation

Recovery of infectious virus from clinical samples was performed using 24-well plates of Vero-76 cells [23]. Plates were seeded 24 h before with 2.5 × 10^5^ cells/well to give 90% confluency. Before infection, plates were washed once with 1 mL of PBS per well; then, 300 μL of AMEM (Wisent Inc., Saint-Jean-Baptiste, QC, Canada) supplemented with 5 μg/mL each of hepes (REF#15630-080, Gibco^TM^, Fisher Scientific Canada), glutaMAX (REF#35-050-061, Gibco^TM^, Fisher Scientific) and gentamycin (REF#15750-060, Gibco^TM^, Fisher Scientific) was added per well. Clinical samples were homogenized, clarified by centrifugation, and inoculated (200 μL sample/well) onto cells. After a one-hour absorption step at 37 °C, an additional 500 µL of AMEM (Wisent Inc.) supplemented with hepes, glutaMAX, gentamycin (5 μg/mL), and 4% FBS (Gibco^TM^, Fisher Scientific) was added to each well. Plates were incubated for 2 days at 37 °C and checked every 24 h for evidence of cytopathic effect.

### 2.9. Serum Neutralization Assay

Testing for neutralizing antibodies was conducted as previously described [11]. Briefly, test sera were heat-inactivated at 56 °C for 30 min and then twofold serially diluted in Alpha-MEM medium (25 µL/well). Two replicates of each dilution were considered for the test. An equal volume (25 μL) of 1000 ID_50_ of VSIV (Indiana Lab strain catalog#VR-3340, ATCC, Manassas, VA, USA) was added to each well of the 96-well tissue culture microtiter plate, except for the wells used for serum toxicity testing. The plates were incubated for 1 h at 37 °C followed by the addition of Vero-76 cells (100 µL/well) in Alpha-MEM medium. The plates were incubated at 37 °C in a 5% CO_2_ incubator, and CPE was scored after 72 h. Serum titers were defined as the reciprocal of the highest dilution producing a 100% inhibition of CPE in both replicates. Back titrations of the challenge virus and positive and negative serum controls were employed to assess test performance. A titer of ≥1:45 of the final dilution was regarded as positive.

### 2.10. Sequencing

The presence of the nucleotide insertion in the intergenic G-L region in the strain IN0919WYB2 was confirmed by Sanger sequencing using the following primers: G1206F-5′-ATTGGACATGGTATGTTGGA-3′ L193R-5′-GAGGGAATCGGAAGAGAATT-3′. RT-PCR was performed using a qScript XLT One-Step RT-PCR kit (REF#CA89409-168, Quantabio, VWR, Edmonton, AB, Canada). Amplification conditions were 48 °C for 20 min; 94 °C for 3 min; followed by 35 cycles of 94 °C for 20 s, 52 °C for 30 s, and 68 °C for 60 s, with a 10 min extension at 68 °C. PCR products were purified using the QIAquick PCR purification kit (REF#28104, Qiagen, Germantown, MD, USA), and Sanger sequencing was carried out using BigDye Terminator V3.1 Cycle Sequencing kit (Applied Biosystems, Thermo Fisher Scientific). Sequencing reactions were run on a 3500xL genetic analyzer (Applied Biosystems, Thermo Fisher Scientific). Raw data were analyzed using Geneious Prime version 2021.0.3 (Biomatters Ltd., Boston, MA, USA).

The identity of 98COE and IN0919WYB2 in the viral stocks used for inoculation was confirmed by next-generation sequencing (NGS). NGS was also used to analyze the isolation positives from the clinical samples. NGS was conducted as follows: DNase treatment (Ambion^TM^, Thermo Fisher Scientific) was performed on the extracted RNA, followed by cleanup with a RNeasy MinElute kit (Qiagen). cDNA was prepared using SuperScript IV (Thermo Fisher Scientific) with Endoh random hexamers (IDT) and VSV-specific primers (VSV_junction_S_IND_1_REV-5′-TTATCCATGATATCTGTTAGTTT-3′ and VSV_junction_S_IND_2_REV-5′-CTGTCCATGATCTCTGTTAGTTT-3′) (IDT) followed by second strand synthesis with NEBNext Ultra II (New England Biolabs, Ipswich, MA, USA) using the manufacturer’s recommended protocol. Double-stranded cDNA was purified using AMPure XP Beads (Beckman Coulter Life Sciences, Indianapolis, IN, USA). Libraries from the purified cDNA pool were made using Nextera XT DNA Library Preparation Kit (Illumina, San Diego, CA, USA) and Nextera XT Index Kit v2 (barcodes) (Illumina) following the manufacturer’s protocol using 20 cycles of PCR during Indexing. An Agilent TapeStation 4200 was used for quality control of the Nextera XT Libraries. Once the library pool was analyzed on the Agilent and the concentration was measured with the Qubit HS DNA kit, the library prep was denatured following the Illumina method. Afterward, libraries were sequenced on the Illumina MiSeq. The resulting fastq files generated by MiSeq were analyzed via the following pipeline.

Preliminary virus detection and assembly were performed with the CFIA-NCFAD/nf-villumina (v2.0.1) Nextflow pipeline [24,25]. First, nf-villumina removed Illumina PhiX Sequencing Control V3 reads using BBDuk (v38.96) [26], followed by adapter removal and quality filtering with fastp [27]. Filtered reads were retained for de novo assembly with Unicycler (v0.4.8) [28], Shovill (v1.1.0) [29], and MEGAHIT (v1.2.9) [30], and the resulting contigs from each assembly were queried against the online NCBI BLAST nucleotide database (accessed 11–16 May 2023 and 30 October 2023) [31]. The fastp filtered reads were mapped to the top VSIV BLAST match for each sample in Geneious Prime version v2023.0.1 [32] with the Minimap2 (v2.24) [33] assembler on default settings. A 75% majority consensus sequence was called with a low coverage threshold of 10× coverage. The resulting consensus sequence was aligned with the de novo assembled contigs with MAFFT (v7.490) [34] to manually check for assembly errors and then checked for the presence of complete coding sequences for the polyprotein gene in Geneious Prime (v2023.0.1) with the Find ORFs tool.

### 2.11. Statistics

The Welch’s *t*-test [35] was used to test the (null) hypothesis that samples evaluated by qRT-PCR derived from two experimental groups have equal means at specific time points post-infection. In this sense, *p*-values < 0.05 were considered significant to reject the null hypothesis. Evaluations were conducted on the statistical discovery software JMP Pro version 16.0.0.

The Pearson correlation analysis [36] was used to assess the strength of the linear relationship between CT values in the sample recovered from nasal and oral swabs and the proportion of viral isolates recovered at specific CT values. Values can oscillate between −1 (total negative linear correlation) and 1 (total positive correlation). Zero indicates no correlation. Analysis was conducted using the statistical software GraphPad version 10.4.0.

## 3. Results

### 3.1. Genomic Analysis

**Figure 3:**
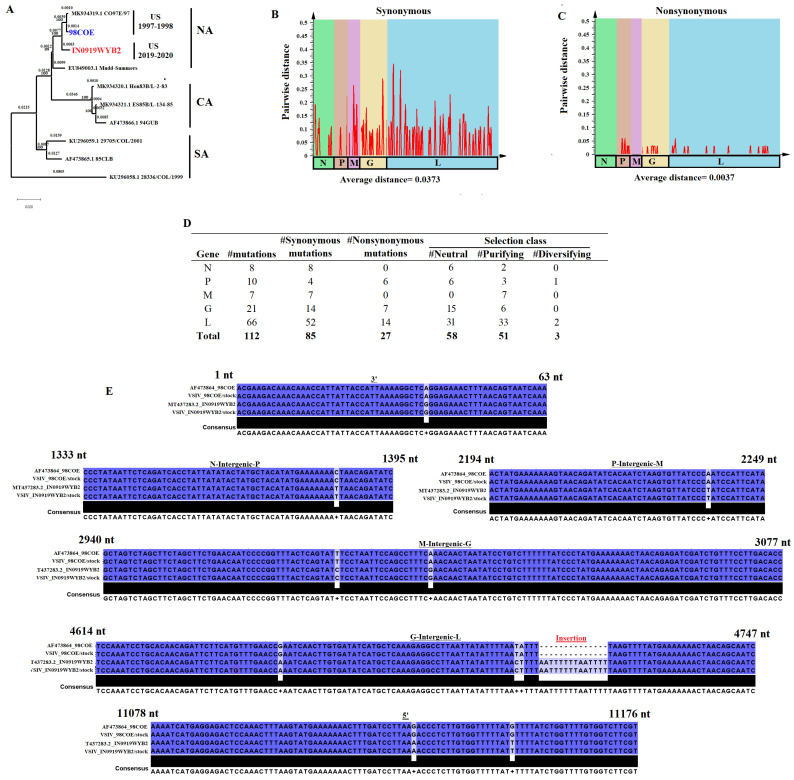
Genomic characterization between the VSIV epidemic strains 98COE and IN0919WYB2. (**A**) Phylogenetic analysis showing the genetic relationship between 98COE and IN0919WYB2. The analysis was conducted using representative VSIV strains from genetic origins in North America (NA), Central America (CA), and South America (SA). The phylogenetic tree was produced using MEGA version 10.2.5. (**B**,**C**) Pairwise distance analysis between 98COE and IN019WYB showing the genetic distance produced between both strains through the accumulation of synonymous and nonsynonymous mutations at different genes. Bars in the graphics represent pairwise distance using a window of 50 nucleotides, with a step of 25. The graph was produced using SSE version 1.2. (**D**) Summary of the distinctive number of synonymous and nonsynonymous mutations between 98COE and IN019WYB within the different genes of VSIV. The selection class reflects a summary of the evolutionary dynamics at different codon sites in each gene where distinctive mutations were predicted. The selection class at different codons was obtained from a previous study assessing the evolution in natural populations of VSIV [3]. (**E**) Location of mutations and insertion at non-coding genome regions of VSIV between 98COE and IN019WYB strains. To enhance the analysis, the original sequences of these viruses previously published in the GenBank database were included (AF473864_98COE and T4337283.2_IN0919WYB2). Nucleotide (nt) boundaries between non-coding regions are based on sequence T4337283.2. Analysis was conducted using the software Jalview version 2.11.1.4.

A detailed genomic analysis was conducted to determine the genomic differences between the VISV strains 98COE and IN0919WYB2 (Figure 3). Phylogenetic analysis of 98COE and IN0919WYB2 along with strains from distinct geographical origins (sequences obtained from Genbank) showed the close phylogenetic relationship between the two (Figure 3A). Strains 98COE and IN0919WYB2 are grouped in the North American cluster (NA) and are phylogenetically distant from the viral strains circulating in Central and South America. Pairwise distance analysis comparing the full-length sequence of both strains indicated an overall nucleotide identity of 98.87%. A total of 121 distinct mutations were observed between these strains (Figure 3B–E). Of these, 112 were located in coding regions, with 85 classified as synonymous and 27 as nonsynonymous. Pairwise distance analysis conducted at either synonymous or nonsynonymous sites indicated that most of the phylogenetic differences between both strains could be explained by the genetic distance produced by the accumulation of synonymous mutations, which was ten times higher than the distance created by nonsynonymous mutations (Figure 3B,C). Furthermore, the analysis revealed a contrasting distribution pattern of mutations, showing that the nonsynonymous mutations were confined to genes P, G, and L (Figure 3B,C).

The evolutionary dynamics were tracked using previously published data to identify potentially relevant mutations on the epidemic VSIV lineage affecting the US between 2019 and 2020 [4]. Based on this information, 109 of 112 distinctive mutations between both strains may be associated with codons evolving under neutral and purifying selection at multiple genes (Figure 3D). Conversely, only three mutations were linked to codons evolving under diversifying selection, indicating that mutations at these codon sites may represent potential phenotypic differences between both strains (Figure 3D). These mutations involve specific codon sites in the P (CGA_(R)_-239-CTA**_(L)_**) and L (CCA_(P)_-1622-CTA_(L**)**_), (CGG(R)-1748-CAG_(Q)_) genes. Detailed information about all differential mutations between both strains can be found in Figure S1. Furthermore, no differences were found between both strains in the genome sequences that encode the predicted C’ and C proteins.

A total of ten characteristic mutations were found distributed among all non-coding genome regions of these strains (Figure 3E). No specific additional procedures were undertaken to determine the sequences in the 3′ and 5′ untranslated regions. Overall, no additional mutations were produced in IN0919WYB2 and CO98 after a single passage on Vero-76 cells to produce the viral stocks used in this study. Moreover, we confirmed the presence of the 14-nucleotide insertion located in the intergenic G-L region of IN0919WYB2 (Figure 3E), a genetic feature distinctive of the epidemic VSIV lineage from the 2019–2020 outbreak [4].

### 3.2. Clinical Assessment

**Figure 4:**
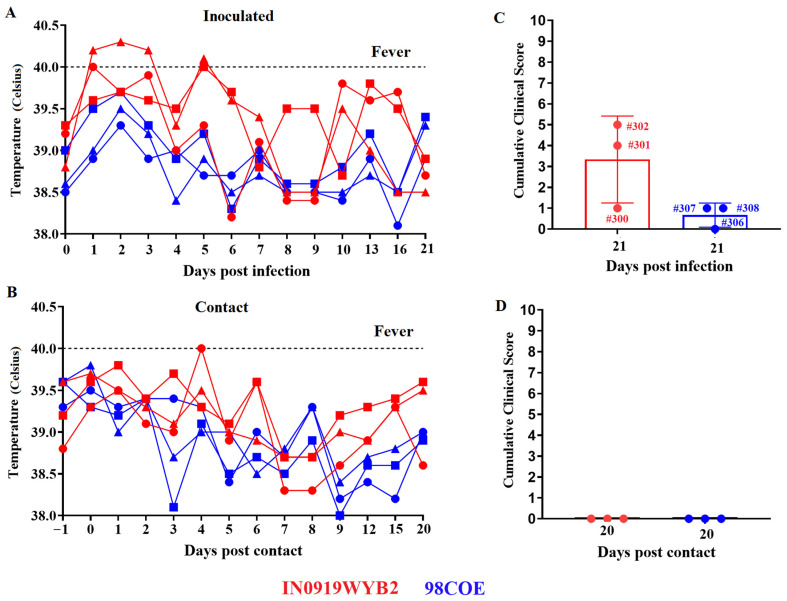
Comparison of clinical outcomes produced by infection with 98COE and IN0919WYB2. Rectal temperatures were monitored on specific days to detect fever in inoculated (**A**) and in contact pigs (**B**) after infection with different viruses. In inoculated pigs, red circles, squares, and triangles denote measurements for pigs 300, 301, and 302, respectively. The same shapes are used for the contact pigs to identify pigs 303, 304, and 305 from the IN0919WYB2 group. A similar pattern is followed for blue shapes (98COE group), identifying pigs 306, 307, and 308 (inoculated) and 309, 310, and 311 (contact), respectively. Final clinical scores were recorded from inoculated (**C**) and contact pigs (**D**) at each group. Scoring key: Pig #300: vesicular lesion in the inoculation site (1 point). Pig #301: vesicular lesions in the hind coronary bands (2 points each one). Pig #302: vesicular lesion in the inoculation site (1 point) and vesicular lesions in the hind coronary bands (2 points each one). Pig #307: vesicular lesion in the inoculation site (1 point). Pig #307: vesicular lesion in the inoculation site (1 point). Graphics were produced using the software GraphPad version 9.5.0.

A pathogenesis study was conducted in pigs to determine potential phenotypic differences between the epidemic VSIV strains 98COE and IN0919WYB2. To assess the severity and dissemination of the disease, we used a clinical scoring system based on the location and number of the vesicular lesions developed during the infection (see Section 2.4). Overall, clinical results suggested that IN0919WYB2 may represent a slightly more virulent phenotype for pigs than the 98COE strain. After inoculation by scarification of the snout, two of three pigs in each group developed vesicular lesions at the inoculation site. In pigs inoculated with IN0919WYB2, lesions were visible in pigs 300 and 302 as early as 2 dpi, while in pigs inoculated with 98COE, lesions were observed at 2 dpi (pig #308) and 4 dpi (pig #307). Fever was observed only in pigs inoculated with IN0919WYB2 (Figure 4A). A temperature of about 40 °C was observed in pigs 300 and 302 at 1 dpi. This febrile response was prolonged in pig #302 until 3 dpi.

Another fever spike was detected again in pigs 301 and 302 at 5 dpi, which correlated with the detection of small vesicular lesions in both hind coronary bands of these pigs at 8 dpi. In the contact group, evidence of fever was recorded only in pig #303 at 4 days post contact (dpc) with the group inoculated with IN0919WYB2 (Figure 4B). Overall, vesicular lesions were not observed in the contact pigs associated with diverse groups. Despite the increased final clinical scores recorded in the group of pigs inoculated with IN0919WYB2 (Figure 4C), no statistically significant differences were observed between the groups. In the case of the contact pigs, final clinical scores were zero for both groups (Figure 4D).

### 3.3. Viral Shedding

**Figure 5:**
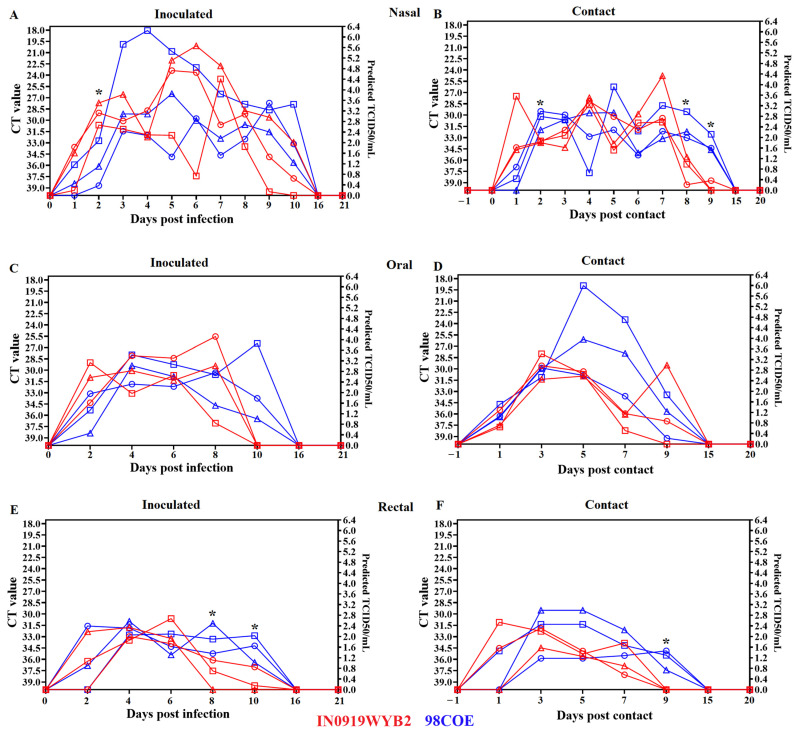
Shedding dynamics produced by infection with 98COE and IN0919WYB2. RT-qPCR was used to evaluate the shedding of viral RNA during infection in nasal, oral, and rectal swabs collected from inoculated ((**A**), (**C**), and (**E**), respectively) and contact ((**B**), (**D**), and (**F**), respectively) animals infected with different viruses. The right *Y*-axis of each graph represents the predicted TCID**_50_**/mL based on the CT value. Predicted levels of infectious virus were considered based on the results of a previous validation [20]. For these graphs, inoculated pigs (IN0919WYB2 group) are represented by red circles, squares, and triangles for pigs 300, 301, and 302, respectively, and to identify contact pigs 303, 304, and 305 (IN0919WYB2 group). Similarly, the same pattern in blue is followed for the 98COE group, identifying pigs 306, 307, and 308 (inoculated) and 309, 310, and 311 (contact), respectively. Asterisks represent time points where means were significantly different between groups (*p*-value < 0.05) by the Welch’s *t*-test.

To evaluate the potential differences in the viral shedding dynamics between 98COE and IN0919WYB2 during infection, RT-qPCR was used to detect the presence of viral RNA in nasal, oral, and rectal swabs. Overall, the results indicated that pigs infected with 98COE shed higher levels of viral RNA for a prolonged period than those infected with IN0919WYB2 (Figure 5).

In nasal swabs, the presence of viral RNA in inoculated animals was detected as soon as 1 dpi, being significantly (*p*-value = 0.0251) higher at 2 dpi in pigs inoculated with IN0919WYB2 (Figure 5A). After that, disparate levels of RNA were detected in inoculated animals within the groups. In the group infected with 98COE, pig #307 displayed RNA levels between days 3 and 5 dpi that reached 5.6–6.4 predicted TCID_50_/mL. Similarly, pig #302, inoculated with IN0919WYB2, showed high RNA levels between 5 dpi and 7 dpi. These levels ranged from 4.8 to 5.6 predicted TCID_50_/mL (Figure 5A).

In the contact animals (Figure 5B), positive samples were detected at 1 dpc, significantly (*p*-value = 0.0150) higher in contact animals infected with IN0919WYB2. However, significantly higher amounts were detected in pigs infected with 98COE at 2 dpc (*p*-value = 0.0230), 8 dpc (*p*-value = 0.0222), and 9 dpc (*p*-value = 0.0019). At 9 dpc, only one (pig #303) of three contact pigs in the IN0919WYB2 group showed some evidence of viral RNA, indicating that viral shedding was more prolonged in contact pigs infected with 98COE. Viral RNA was not detected beyond 9 dpc in either group.

Overall, both groups observed no statistically significant differences in oral shedding (Figure 5C,D). However, a prolonged excretion of viral RNA was recorded in pigs inoculated with 98COE (10 dpi) compared with pigs inoculated with IN0919WYB2 (8 dpi) (Figure 5C). No differences were observed in the duration of oral shedding for contact pigs. Nevertheless, at 5 and 7 dpc, increased amounts of viral RNA were detected in pigs 310 and 311, ranging between 3.5 and 6.0 predicted TCID_50_/mL.

Finally, viral shedding was assessed in rectal swabs. In inoculated animals, significantly higher levels of RNA were found in pigs inoculated with 98COE at 8 dpi (*p*-value = 0.0465) and 10 dpi (*p*-value = 0.03252) (Figure 5E). Similarly, increased RNA levels were observed in 98COE contact animals at 9 dpi (*p*-value = 0.0059) (Figure 5E). In contact animals, we also observed an extended viral shedding in animals infected with 98COE (Figure 5E).

No viral RNA was detected in the blood or serum samples from any pig in either experimental group.

### 3.4. Detection of Infectious Virus

**Figure 6:**
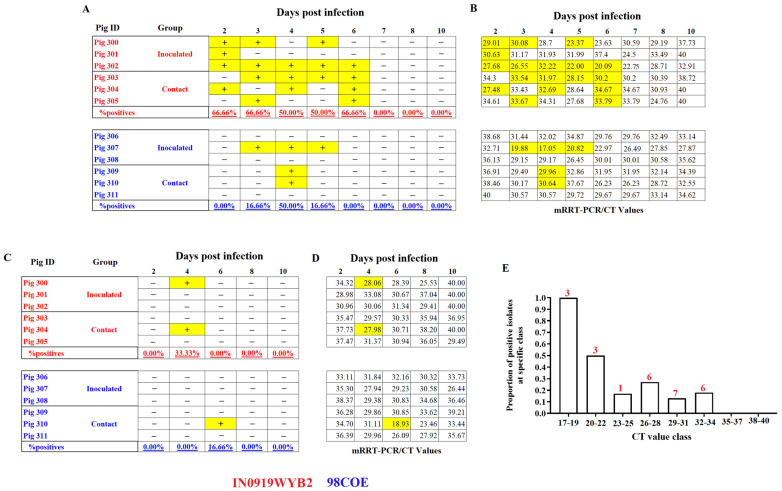
Analysis of the recovery of infectious viral particles from nasal and oral swabs during infection with 98COE and IN0919WYB2. (**A**) Differences in the recovery of infectious virus from nasal swabs between pigs infected with 98COE and IN0919WYB2. (**B**) CT values obtained from the same nasal swabs subjected to viral isolation. (**C**) Differences in the recovery of infectious virus from oral swabs between pigs infected with 98COE and IN0919WYB2. (**D**) CT values obtained from the same oral swabs subjected to viral isolation. Specific days and pigs where viral isolations were obtained are highlighted in yellow in all cases. (**E**) Proportion of positive viral isolates using nasal and oral swabs at specific CT value ranges. The number of isolates obtained for each CT group is denoted by the numbers (in red) on the top of the bars.

After assessing differences in RNA shedding by RT-qPCR from clinical samples collected during the experiments, we attempted to understand if the differences in shedding dynamics observed between both groups were consistent with the recovery of infectious virus in Vero-76 cells. Despite the increased and prolonged levels of viral RNA shed by pigs infected with 98COE, more infectious VSV was recovered from pigs infected with IN0919WYB2. In particular, viral shedding detected in nasal swabs between 2 dpi and 6 dpi showed that a higher proportion of pigs infected with IN0919WYB2 excreted more infectious virus than pigs infected with 98COE (Figure 6A). Furthermore, this pattern was also characterized by the recovery of infectious virus from all six pigs infected with IN0919WYB2, contrasting with the detection of infectious particles in just three pigs of the group infected with 98COE (Figure 6A). Overall, despite viral RNA in nasal swabs, infectious virus was not recovered beyond 6 dpi (Figure 6B).

In oral swabs, minimal differences were observed between groups. At 4 dpi, infectious virus was recovered exclusively from two pigs in the group infected with IN0919WYB2, while a single viral isolate was obtained from the group infected with 98COE at 6 dpi (Figure 6C). Like the pattern observed in nasal swabs, despite CT values, no infectious virus was recovered beyond 6 dpi (Figure 6D). The minimal detection of infectious viral particles in both groups clearly suggested that the shedding of infectious particles from the oral cavity was not an important source of infectious virus induced during the infection with these two strains. No viral isolates were obtained from either animal group’s rectal swabs.

Due to the apparent lack of correlation between the recovery of infectious virus and the presence of CT values, a Pearson r correlation analysis was performed. This analysis indicated a significant negative correlation (*p*-value = 0.0081, r = −0.8459) between the CT value in the sample and the proportion of viral isolates recovered at specific CT values (Figure 6E). In samples with CT values between 17 and 19, the percentage of infectious particles recovered was 100%. However, above this range of CT values, there was a decrease in the recovery of infectious viruses (Figure 6E). Overall, 73% (*n* = 19) of the viral isolates obtained in both groups were associated with CT values between 26 and 34 (Figure 6E).

Additionally, a DAS ELISA was performed to infer if the lack of viral isolates observed in RT-qPCR positive samples was associated with the absence of viral antigens, denoting the presence of viral RNA in the clinical samples only. First, using several ten-fold dilutions of the viral stocks 98COE and IN0919WYB2, we determined the limit of detection of the DAS ELISA for both antigens. RT-qPCR was performed for each dilution. Limits of detection associated with CT values of 21.29 (1 × 10^5.43^ TCID_50_/mL) and 22.26 (1 × 10^6.00^ TCID_50_/mL) were observed for IN0919WYB2 and 98COE, respectively (Figure S2A,B). Overall, all nine of the viral isolates recovered during the experiment used as a control came positive by DAS ELISA, indicating the ability of this test to detect viral particles recovered from pigs (Figure S2C). In this sense, based on the detection limit predicted for the DAS ELISA, a total of six samples with CT values ranging between 22.75 and 26.49, all negative by viral isolation, were evaluated by DAS ELISA. Positive results by DAS ELISA were obtained in three (CT values 22.97, 23.46, and 24.76) of the six samples (Figure S2C), indicating the presence of viral antigens in some of these samples. Two of these samples came from nasal swabs from the inoculated pig #307 (98COE group) and from the contact pig #305 (IN0919WYB2 group) collected at dpi 6 and 8, respectively. The last sample was an oral swab collected at 8 dpi from the contact pig #310 (98COE group).

### 3.5. Postmortem Sample Evaluation

**Figure 7:**
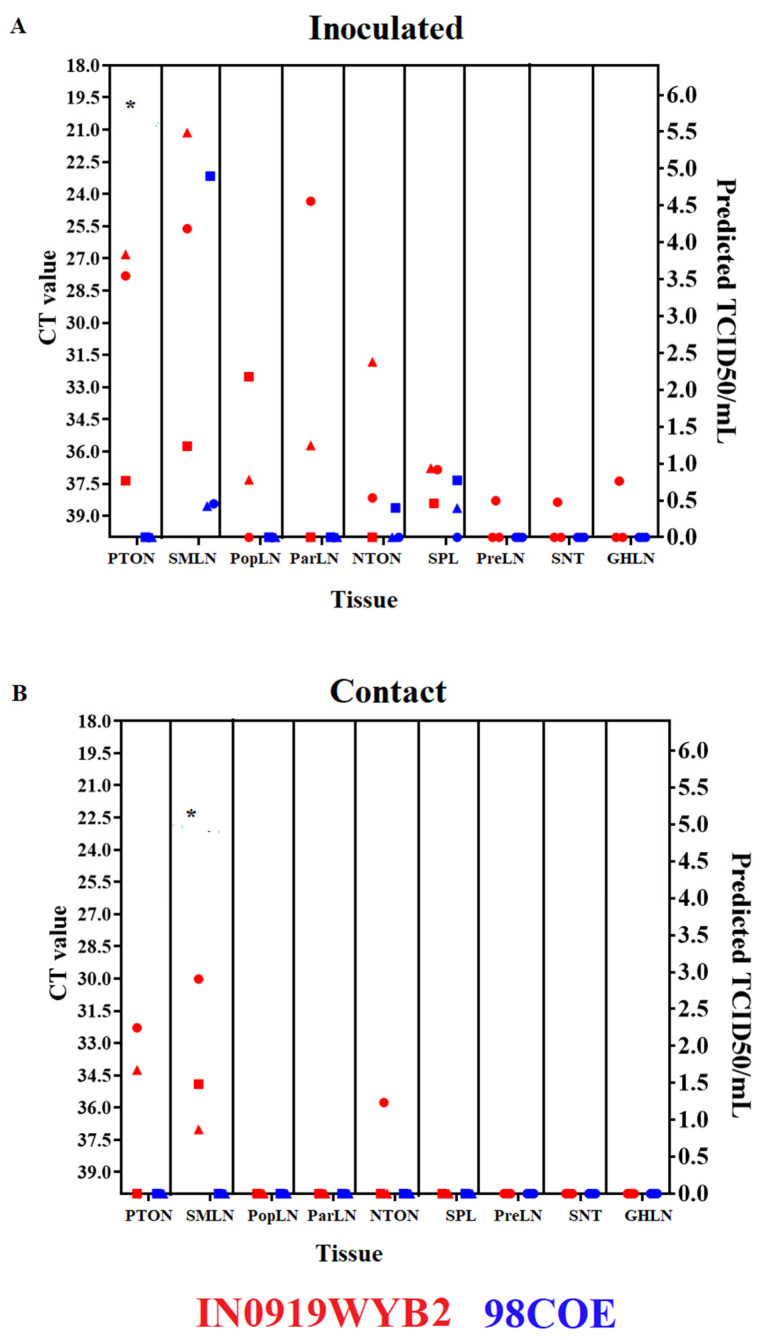
Postmortem evaluation between 98COE and IN0919WYB2. At 21 dpi, necropsies were conducted in inoculated (**A**) and contact (**B**) pigs from each group. Tissues were evaluated by RT-qPCR. The right *Y*-axis reflects the predicted TCID_50_/mL based on the CT value. Abbreviations indicate tonsil of the soft palate (PTON), submandibular lymph node (SMLN), popliteal lymph node (R-PopLN), parotid lymph node (ParLN), nasopharyngeal tonsil (NTON), spleen (SPL), prescapular lymph node (PreLN), and gastrohepatic lymph node (GHLN). IN0919WYB2 group pigs identified by red circles, squares, and triangles denote measurements of pigs 300, 301, and 302, respectively. Identical shapes are used for contact pigs to identify pigs 303, 304, and 305. A similar pattern is used to denote the 98COE group, with blue shapes identifying pigs 306, 307, and 308 (inoculated) and 309, 310, and 311 (contact), respectively. Asterisks represent tissues where means were significantly different between groups (*p* < 0.05) by the Welch’s *t*-test.

A total of 12 postmortem tissues were collected from all pigs in each group at 21 dpi to evaluate potential differences in the viral distribution in tissues between pigs infected with either 98COE or IN0919WYB2. Overall, higher levels of viral RNA, based on RT-qPCR CT values, were observed in multiple tissues in pigs infected with IN0919WYB2 versus 98COE (Figure 7). In inoculated pigs, significantly (*p*-value = 0.04) higher levels of viral RNA were found in the tonsil of the soft palate in animals inoculated with IN0919WYB2, contrasting with pigs inoculated with 98COE, where no evidence of viral RNA was found in this tissue (Figure 7A). Furthermore, while no viral RNA was present in the popliteal, parotid, prescapular, or gastrohepatic lymph nodes or in the snout skin from animals inoculated with 98COE, evidence of viral RNA was found in one of two inoculated animals from the IN0919WYB2 group (Figure 7A). Evidence of viral RNA was found in the popliteal lymph nodes of animals 301 and 302, correlating with the presence of vesicular lesions in both hind coronary bands of these pigs and confirming the ability of IN0919WYB2 to disseminate away from the inoculation site.

In contact animals, viral RNA was undetectable in the tissues of the 98COE group (Figure 7B). In contrast, significantly (*p*-value = 0.04) higher levels of viral RNA were observed in the submandibular lymph nodes of contact pigs in the IN0919WYB2 group. In these animals, the presence of viral RNA was also detected in two pigs in the tonsil of the soft palate and one pig in the nasopharyngeal tonsil (Figure 7B).

No viral RNA from either group was detected in the following: neck skin, anterior tongue epithelium, or liver. In all cases, no infectious isolates were obtained from either group. However, based on the potentially high concentrations of predicted viral titers (predicted TCID_50_/mL based on CT values) (Figure 7A), we decided to look for the presence of viral antigens. Viral antigen was detected in inoculated animal #300 in the submandibular and parotid lymph nodes (corrected ODs 0.227 and 0.183, respectively) and in #307 in the submandibular lymph node (corrected OD 0.193), showing the presence of viral antigen at 21 dpi in both infected groups.

### 3.6. Neutralizing Antibody Response

**Figure 8:**
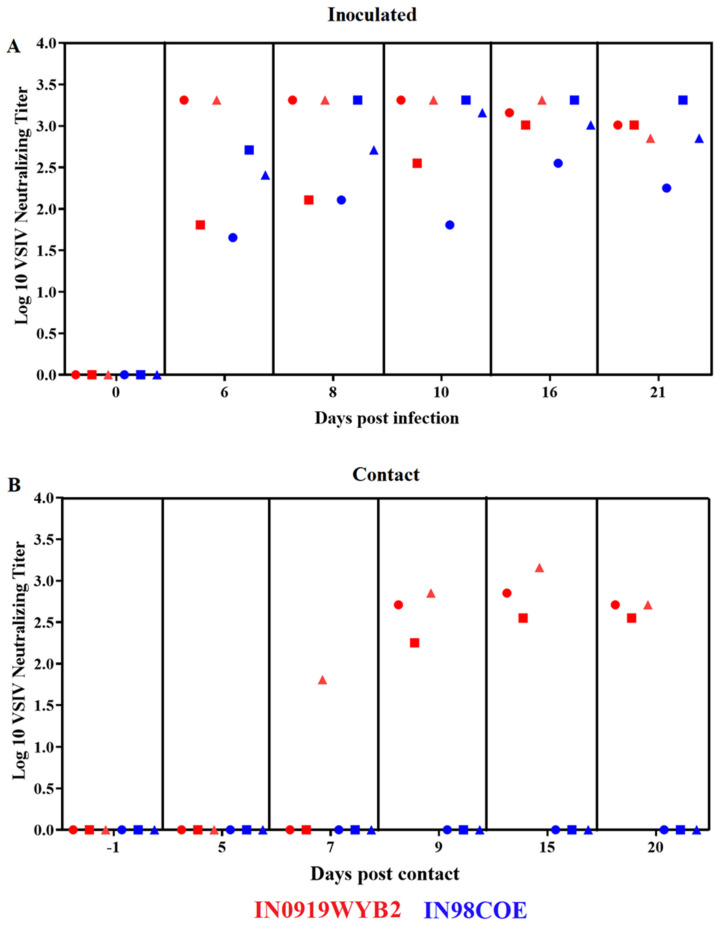
Differences in the adaptive immune response between 98COE and IN0919WYB2 groups during the infection in pigs. Graphics show the titer of neutralizing antibodies against VSIV at different times in inoculated (**A**) and contact (**B**) pigs infected with 98COE or IN0919WYB2. In inoculated pigs, red circles, squares, and triangles denote measurements from pigs 300, 301, and 302, respectively. The same shapes are used for contact pigs to identify pigs 303, 304, and 305 from the IN0919W9YB2 group. Similarly, the blue shapes (98COE group) identify pigs 306, 307, and 308 (inoculated) and 309, 310, and 311 (contact), respectively.

To assess potential differences in the adaptive immune response produced by the infection of pigs with either 98COE or IN0919WYB2, serum samples collected during the experiment were tested for virus neutralization. Overall, no differences were observed between inoculated animals from the different groups (Figure 8A).

Overall, at 6 dpi, neutralizing antibodies were recorded in all inoculated animals of both groups. The presence of neutralizing antibodies was consistent until the end of the experiment (Figure 8A). However, a dissimilar pattern was observed in contact animals between the groups. While the presence of neutralizing antibodies in contact pigs from the IN0919WYB2 group was evident between 7 and 9 dpc, all contact pigs from the 98COE group failed to develop neutralizing antibodies during the time of the experiment (Figure 8B). This lack of neutralizing antibody response clearly correlates with the absence of viral RNA in multiple tissues collected from contact pigs in the group 98COE (Figure 7B), providing evidence of the absence of contact transmission for 98COE.

### 3.7. Evolutionary Dynamics of 98COE and IN0919WYB2 During Infection in Pigs

**Figure 9:**
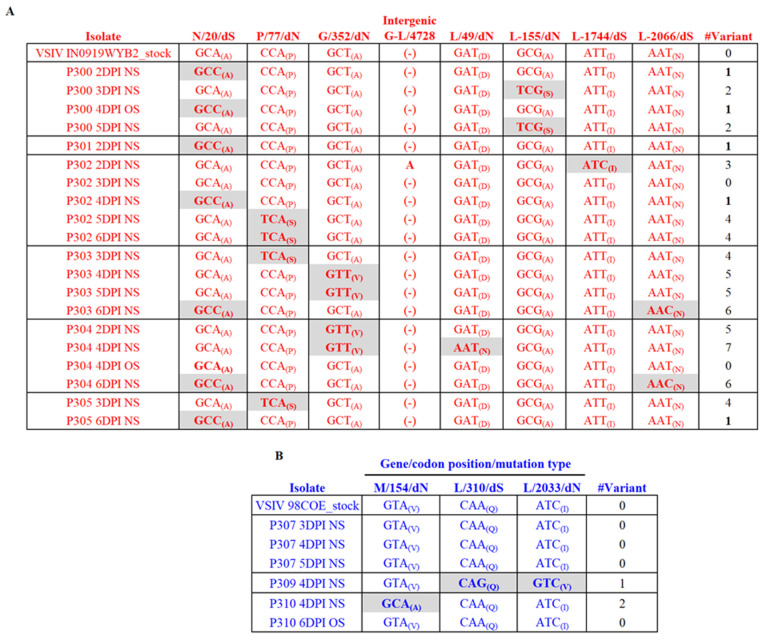
Genetic characteristics of the viral progeny produced during the infection of IN0919WYB2 and 98COE in pigs. To assess the viral progeny diversity produced during the infection with strains IN0919WYB2 (**A**) and 98COE (**B**), viral isolates recovered from pigs infected with each of the strains were subjected to next-generation sequencing (NGS). Mutations at different codons were identified by gene/codon position and mutation type (dS = synonymous, dN = nonsynonymous substitutions). Gray highlights denote substitutions at specific codon positions compared with parental phenotype in the viral stock. Letters in parentheses reflect the amino acid encoded by different codons.

Finally, we explored potential differences between 98COE and IN0919WYB2 regarding their ability to induce viral variants during the infection in pigs. Our results showed that inoculation with IN0919WYB2 resulted in more genetic variability during the infection in pigs compared with 98COE (Figure 9). Overall, a total of seven single nucleotide polymorphisms (SNPs) were found distributed in genes N, P, G, and L among viruses recovered from the pigs infected with IN0919WYB2, half of which were associated with nonsynonymous changes (Figure 9A). Additionally, an insertion was found in the intergenic G-L region from pig #302 (Figure 9A), which corresponds to the addition of an extra A in the highly conserved seven adenine polyadenylation signal present in the intergenic regions of VSV [37]. Our sequencing analysis revealed an interesting infection dynamic pattern, where seven viral variants at the consensus level were identified during the infection of pigs with IN0919WYB2 (Figure 9A). The consensus sequence associated with the viral stock (variant #0) was observed in only 2 of 20 viral isolates recovered during the infection, indicating that multiple viral variants dominated the infection dynamics in this experimental group. Specific changes at codon positions N/20, P/77, G/352, and L/2066 were observed in different variants, affecting multiple pigs at different time points during the infection. In this context, variant #1 was the most prevalent, identified in three pigs at multiple dpi (Figure 9A).

The synonymous mutation GCA-GCC in gene N/codon 20 was the most prevalent mutation during infection and was observed in seven isolates involving variants #1 and #6. It was the only mutation observed in the isolates recovered from all pigs at specific time points during the infection (Figure 9A). Additionally, in pig #304 at four dpi, two different variants (#0 and #7) were recovered from oral and nasal swabs, respectively (Figure 9A). The diverse nature of the mutations observed does not preclude the reversion back to the original state (Figure 9A). Conversely, in pigs infected with 98COE, the infection was dominated by variant #0 (Figure 9B). In this group, two different variants were identified (Figure 9B), one in pig #309 at 4 dpi and the other in pig #310 at 4 dpi. In pig #310, we observed the same pattern described in the other group, where the original viral phenotype was recovered later during the infection (Figure 9B).

## 4. Discussion

Recently, the epidemic 2019–2020 VSIV lineage IN0919WYB2 emerged in the US, producing one of the largest outbreaks in the country [2]. The epidemiological characteristics of this lineage starkly differ from those of the previous VSIV lineage that impacted the US during 1997–1998 [10], suggesting potential phenotypic distinctions between the two. In this scenario, similar to observations in other well-documented arboviruses like the Venezuelan Equine Encephalitis Virus [38], it is uncertain whether the emergence of these epidemic lineages could be associated with the ability of specific viral phenotypes to be more efficient at replicating in either vertebrate hosts or vectors or even during outbreaks.

In this study, pigs, a natural host of VSV [8,11,16,17], were used to determine phenotypic differences in the vertebrate host between the strains 98COE and IN0919WYB2 associated with the epidemic lineages affecting the US in 1997–1998 and in 2019–2020, respectively. Since a previous study showed the host predilection of different VSV strains [39], a possible concern in the interpretation of the results from our study is the distinct host origin of 98COE (horse) and IN0919WYB2 (bovine). In this sense, it has been shown that pigs represent a neutral host model for pathogenesis studies, being unaffected by the host predilections seen in some VSNJV strains from bovine origin. However, at this point we cannot rule out the possible effect in the pathogenesis in pigs produced by the strain 98COE. Future studies are needed to determine the neutrality of pigs as a model for pathogenesis studies from strains of horse origin.

Multiple genomic differences between 98COE and IN0919WYB2 may be potentially linked to the phenotypic differences observed in this study. Indeed, one of the most remarkable differences between both strains was the 14-nucleotide insertion located in the G-L intergenic region of IN0919WYB2. The presence of insertions in the G-L intergenic region has been described in VSIV strains from North American and Central American origin [4,40]. At this point, we do not have an explanation regarding the possible role of this insertion in the phenotype displayed by IN0919WYB2 in pigs. Our results are consistent with a previous pathogenesis study in pigs [9] showing that VSIV strains from a Central American origin carrying long insertions in the intergenic G-L region appeared more virulent than VSIV strains that originated in North America without insertions, suggesting the potential role of this insertion as a promoter of virulence in VSIV. Future experiments using reverse genetics are needed to acquire more insights about the possible role of the insertion in the phenotype observed in IN0919WYB2 infections in pigs.

Furthermore, our genomic analysis identified multiple putative mutations between both strains. Among these, three mutations at gene/codons P (CGA_(R)_-239-CTA_(L**)**_) and L (CCA(P)-1622-CTA_(L**)**_), (CGG_(R)_-1748-CAG_(Q)_) previously found under positive selection on natural populations of VSIV [4], might be considered to explain the phenotypic differences between both viruses. However, since analysis to identify the evolutionary significance of these mutations was conducted in silico [4], future studies using reverse genetics are needed to assess the possible role of these mutations in the pathogenesis of VSIV.

Previously, the epidemic VSNJV NJ0612NME6 was characterized, resulting in a highly virulent phenotype in pigs when compared with its closet endemic ancestor (NJ0806VCB) [11], suggesting that increased virulence is a hallmark of the epidemic VSV lineages. However, clinically, our results were consistent with previous publications, indicating the overall low virulence displayed by VSIV strains in pigs [8,41,42], even though the strains used in our study were associated with epidemic lineages. In our study, it was impossible to establish any statistically significant difference in terms of virulence between the strains based on the development of vesicular lesions. However, based on the development of small secondary vesicular lesions in two of three pigs infected with IN0919WYB2, it is possible to hypothesize that this virus may represent a slightly more virulent phenotype for pigs than 98COE. On the other hand, the fact that just two of three inoculated pigs at each group developed vesicular lesions in the inoculation site in the snout strongly suggests that both strains have similar levels of infectivity.

Regarding the evaluation of viral RNA in clinical samples, our results contrast with a previous study in pigs, where viral RNA in nasal and tonsil swabs was inconsistently found in infected animals [9]. Conversely, our results were consistent with previous studies on VSNJV using RT-PCR [11,16,17], showing shedding of viral RNA occurring as early as 1 dpi and 1 dpc in all pigs. A finding in our study was the lack of correlation between the presence of viral RNA in clinical samples and the recovery of infectious viruses. Despite detecting viral RNA in both groups, live virus, especially from nasal swabs, was mainly recovered from pigs (inoculated and contact) infected with IN0919WYB2. This observation highlights the efficiency of IN0919WYB2 to produce infectious particles during the infection in pigs. In this context, as seen in other viruses like Crimean–Congo hemorrhagic fever virus and yellow fever virus [43], our results make it possible to hypothesize that IN0919WYB2 might be more efficient than 98COE in synchronizing the genome and particle production. In terms of transmissibility, this ability might be considered a critical difference between the strains evaluated in our study. Future studies are needed to assess potential differences between both viruses in terms of infectiousness.

To find a possible explanation for the lack of correlation between viral RNA and infectious virus seen in multiple clinical samples, we attempted to detect the presence of viral antigen using the DAS ELISA. However, because of the low sensitivity showed by this assay, the presence of viral antigen was confirmed in just three of the six clinical samples that tested negative for the presence of infectious particles from three distinct pigs. Despite the limited number of samples detected by DAS ELISA, it is possible to propose some conclusions. Two of the samples, from pigs #305 and #307, were collected at 8 dpi and 6 dpi, respectively, when the levels of neutralizing antibodies were between 2.5 and 3.5 log 10 VSIV neutralizing titer, suggesting that the presence of antibodies was responsible for our failure to isolate infectious particles from these clinical samples and others collected later during the infection. However, based on the positive sample detected from pig #310 at 8 dpi in the absence of neutralizing antibodies, we may suggest the potential presence of defective interfering virus particles (DIVPs). Additionally, three samples collected from postmortem tissues at 21 dpi from two pigs were also found positive by DAS ELISA, indicating that VSIV may persist in pigs after an acute infection. This result is consistent with a study showing the existence of a prolonged antigen presentation after an acute infection in mice, potentially contributing to an ongoing immune response [44]. Future studies are needed to understand this finding in pigs.

DIVPs are well known for their properties to modulate virulence by triggering the innate immune response and interfering with viral replication [45]. In our study, the lack of correlation between viral RNA and infectious virus was more evident in the group of pigs infected with the 98COE strain, suggesting that increased levels of DIVPs might have been produced during the infection of pigs with this strain and, therefore, providing a possible hypothesis for the reduced levels of virulence seen in this experimental group. Our results may be consistent with a previous study, which found that there was cycling production of infectious virus in the brains of mice infected with VSV [46], a condition wherein there is alternating dominance between infective and interfering viral particles during the infection. In other arboviruses like the Zika virus, it has been demonstrated that DIVPs are antiviral in vivo, in both mice and mosquito vectors, reducing the transmission in the vector by up to 90% [47]. Further experiments are needed to probe the potential relevance of DIVPs during the infection of VSV in pigs.

A postmortem lymph node evaluation was performed in our study to contrast the ability of both strains to disseminate along the seven lymphatic territories previously described in pigs (parotid, mandibular, dorsal cervical, ventral cervical, subiliac, inguinal, and popliteal) [48]. Overall, in contrast to the group of pigs inoculated with 98COE, the positive results in the PTON and SLMN lymph nodes in all three pigs inoculated with IN0919WYB2 suggest the ability of this strain to replicate at higher levels in the parotid and mandibular lymphatics, respectively. This observation was consistent with the higher levels of infectious virus recovered from pigs infected with IN0919WYB2, supporting that this strain may represent a more virulent phenotype than 98COE for pigs. Furthermore, considering the inoculation route used in this study and the presence of positive samples in the PopLN (popliteal territory) in two of three pigs inoculated with IN0919WYB2, it can also be suggested that IN0919WYB2 has the potential to disseminate systemically during infection, which may reflect the claimed increase in virulence of this strain. However, most positive results were found in the parotid (PTON and NTON) and mandibular (SMLN and ParLN) lymphatic territories in both groups, suggesting that during the infection in pigs with these two strains, the majority of viral replication takes place locally, close to the primary site of the infection. This situation highly contrasts with our previous results with VSNJV [11], showing the intrinsic differences between both serotypes to produce a systemic infection in pigs. This situation can be correlated with the severity of the clinical outcomes observed during the infection with VSNJV in pigs [11].

Unlike the contact pigs evaluated from the IN0919WYB2 group, no positive results (for nucleic acid or live virus) were obtained from postmortem samples collected from the contact pigs from the 98COE group. In this context, the limited number of viral isolations obtained from this contact group (n = 1) may reflect the extremely low levels of infectious particles circulating in these pigs, providing a possible explanation not only for the absence of neutralizing antibodies but also the lack of RNA detection in postmortem samples. These results also stress the value of including contact pigs in experimental groups during pathogenesis studies to assess differences in levels of transmissibility among VSIV strains.

Finally, in this study, we evaluated a poorly understood aspect of the evolution of VSV during an in vivo infection in a natural host. Overall, we consider that the results of this approach generated an interesting perspective, not only due to the possible differences in the evolutionary dynamics between the two VSIV epidemic strains but also to an increase in our insights about the potential role of the vertebrate host during the infectious cycle of VSV in nature. Our results also showed the possible relevance of the vertebrate host in the occurrence of viral diversity during infection, a condition dependent on the intrinsic characteristics of specific viral strains. Based on the above statements, we conclude that the ability of IN0919WYB2 to induce an increased number of viral variants during the infection in pigs might have represented an evolutionary advantage for the VSIV lineage associated with the epidemic outbreak of 2019–2020 in the US. This characteristic may be considered an additional factor to help understand the epidemiological differences recorded between the epidemic VSIV lineages from 1997–1998 and 2019–2020 [2].

In this sense, among the multiple mutations reported during our study, the GCT**_(A)_**-GTT**_(V)_** mutation at codon G/352, associated with a neutralizing epitope [49], was the only mutation found that was previously reported in VSIV in nature [4]. The report showed that the substitution GCT**_(A)_**-GTT**_(V)_** was a putative mutation linked to the group of viruses dominating the VSIV outbreak in the US during 2020 [4]. Based on the results of our pathogenesis study, we suggest that the emergence of the mutation GCT(A)-GTT_(V)_ during the outbreak was probably the result of an infection in livestock instead of an insect vector. However, because neither this mutation nor the other mutations reported in this study became dominant during the infection in pigs, we speculate that genetic drift instead of natural selection may be responsible for the evolutionary pattern observed in this study, suggesting that the mutation GCT_(A**)**_-GTT_(V)_ might not be implicated in the adaptation of VSV in livestock. In this context, the fact that the mutation GCT_(A)_-GTT_(V)_ was fixed and maintained in the sub-lineage dominating the infections in the outbreak during 2020 after multiple transmission events [4] strongly suggests that this mutation might have represented an adaptive advantage for this sub-lineage to replicate in a specific insect vector during the outbreak. More studies are needed to assess the potential role of the multiple mutations described in this study in viral adaptation in livestock and insect vectors.

In conclusion, our study indicates that the VSIV epidemic strains 98COE and IN0919WYB2, which represent the epidemic lineages of 1997–1998 and 2019–2020, respectively, constitute two different phenotypes in terms of transmissibility for pigs. This phenotypic difference may be correlated with the epidemiological discrepancies observed between the outbreaks produced by both lineages in the US [2,10]. The recovery of infectious virus from contact pigs was obtained from animals in a subclinical state, suggesting that this situation may be a confounding factor during epidemic outbreaks, favoring the transmission of VSIV by the movement of apparently uninfected animals. In this sense, our results make it possible to hypothesize a scenario where in the absence of clinical disease, other sources of infection beyond the vesicular lesions should be considered as a potential source of the infection of susceptible animals or insect vectors. Finally, our results indicate that protocols for pathogenesis studies of VSV should be redefined beyond the typical assessment based on just the detection of vesicular lesions. Some important limitations must be considered in this study. One is the lack of characterization of the specific viral RNA particles detected in the clinical samples (genomic RNA, antigenomic RNA, and messenger RNA) by RT-qPCR. Second is our inability to produce full-length genome sequences directly from clinical samples that were positive by RT-PCR but with an absence of infectious viral particles. We consider that these approaches might have given insights about the lack of correlation observed in this study between viral RNA and infectious virus and the potential relevance of DIVPs during the replication of VSIV in pigs. Additionally, the lack of in vitro characterization to assess possible differences in the gene expression related to the innate immune response between both strains should be considered an additional limitation during this study.

## Data Availability

The sequences generated during this project are available in the NCBI under the accession numbers PQ622883-PQ622910.

## Supplementary Materials

The following supporting information can be downloaded at: https://www.mdpi.com/article/10.3390/v16121915/s1, Figure S1: Differential mutations at multiple gene codon regions between 98COE and IN0919WYB2; Figure S2: Evaluation of clinical samples found positive by RT-qPCR using DAS ELISA.
