# Supplementary material for: Phenotypic Differences Between the Epidemic Strains of Vesicular Stomatitis Virus Serotype Indiana 98COE and IN0919WYB2 Using an In-Vivo Pig (Sus scrofa) Model
Source: Viruses. 2024 Dec 13;16(12):1915. doi: 10.3390/v16121915 (PMC11680245; doi:10.3390/v16121915)
Supplement: Supplementary file 1 [file viruses-16-01915-s001.zip › viruses-3281303-supplementary - final doc.pdf]

viruses

MDPI

Supplementary Materials

Phenotypic Differences Between the Epidemic Strains of Vesicular Stomatitis Virus Serotype Indiana 98COE and IN0919WYB2 Using an In-Vivo Pig (Sus Scrofa) Model

Gene	# Codon	Allele 98COE	Allele IN019WYB2	Type of change	Selection class
N	29	GCG(A)	GCA(A)	Synonymous	Neutral
N	40	CTT(L)	CTA(L)	Synonymous	Neutral
N	48	TTG(L)	CTG(L)	Synonymous	Neutral
N	89	TCA(S)	TCT(S)	Synonymous	Purifying
N	110	CTG(L)	TTG(L)	Synonymous	Neutral
N	114	GAT(D)	GAC(D)	Synonymous	Purifying
N	311	ACT(T)	ACC(T)	Synonymous	Neutral
N	348	TTC(F)	TTT(F)	Synonymous	Neutral
P	112	GAG(E)	AAG(K)	Nonsynonymous	Neutral
P	113	CTC(L)	CTT(L)	Synonymous	Purifying
P	114	AAA(K)	GAA(E)	Nonsynonymous	Neutral
P	161	GCA(A)	ACA(T)	Nonsynonymous	Neutral
P	169	GAA(E)	AAA(K)	Nonsynonymous	Neutral
P	215	CAG(Q)	CCG(P)	Nonsynonymous	Neutral
P	239	CGA(R)	CTA(L)	Nonsynonymous	Diversifying
P	243	AAA(K)	AAG(K)	Synonymous	Purifying
P	248	CTT(L)	CTC(L)	Synonymous	Neutral
P	251	AGG(R)	AGA(R)	Synonymous	Purifying
M	16	AAG(K)	AAA(K)	Synonymous	Purifying
M	102	CGG(R)	CGT(R)	Synonymous	Purifying
M	108	TTG(L)	CTG(L)	Synonymous	Purifying
M	109	GCA(A)	GCT(A)	Synonymous	Purifying
M	135	TGC(C)	TGT(C)	Synonymous	Purifying
M	162	TTC(F)	TTT(F)	Synonymous	Purifying
M	169	GGG(G)	GGA(G)	Synonymous	Purifying
G	11	TCC(S)	TTC(F)	Nonsynonymous	Neutral
G	26	CAA(Q)	CAG(Q)	Synonymous	Neutral
G	41	CCT(P)	CCG(P)	Synonymous	Purifying
G	70	GCT(A)	GCA(A)	Synonymous	Purifying
G	109	AGG(R)	AAG(K)	Nonsynonymous	Neutral
G	124	GGA(G)	GGC(G)	Synonymous	Neutral
G	127	CCT(P)	CCC(P)	Synonymous	Purifying
G	189	GTA(V)	GTC(V)	Synonymous	Purifying
G	200	ATG(M)	ACG(T)	Nonsynonymous	Neutral
G	213	TCC(S)	TCT(S)	Synonymous	Purifying
G	217	GAG(E)	GGG(G)	Nonsynonymous	Neutral
G	241	AAG(K)	AGG(R)	Nonsynonymous	Neutral
G	251	GTC(V)	GTT(V)	Synonymous	Neutral
G	258	CAG(Q)	AAG(K)	Nonsynonymous	Neutral
G	308	GGG(G)	AGG(R)	Nonsynonymous	Neutral
G	335	ATC(I)	ATT(I)	Synonymous	Neutral
G	381	GAC(D)	GAT(D)	Synonymous	Neutral
G	439	TTA(L)	TTG(L)	Synonymous	Neutral
G	445	GGA(G)	GGG(G)	Synonymous	Neutral
G	451	ATC(I)	ATT(I)	Synonymous	Purifying
G	455	GAA(E)	GAG(E)	Synonymous	Neutral
L	15	AAC(N)	AAT(N)	Synonymous	Purifying
L	54	ATT(I)	ATC(I)	Synonymous	Neutral
L	97	AAT(N)	AGT(S)	Nonsynonymous	Neutral
L	109	GGA(G)	GGG(G)	Synonymous	Neutral
L	131	ATC(I)	GTC(V)	Nonsynonymous	Neutral
L	146	AAA(K)	AGG(R)	Nonsynonymous	Neutral
L	149	GAC(D)	GAT(D)	Synonymous	Purifying
L	155	GCT(A)	GCG(A)	Synonymous	Neutral
L	156	TAT(Y)	CAT(H)	Nonsynonymous	Neutral
L	214	GAA(E)	GAG(E)	Synonymous	Neutral
L	279	GGA(G)	GGG(G)	Synonymous	Purifying
L	282	TCT(S)	TCC(S)	Synonymous	Purifying
L	289	GTA(V)	GTG(V)	Synonymous	Purifying
L	333	AAC(N)	AAA(K)	Nonsynonymous	Neutral
L	369	CTG(L)	CTA(L)	Synonymous	Neutral
L	387	GCA(A)	GCG(A)	Synonymous	Purifying
L	388	AAG(K)	AAA(K)	Synonymous	Purifying
L	400	TAT(Y)	TTT(F)	Nonsynonymous	Neutral
L	420	CAT(H)	CAC(H)	Synonymous	Purifying
L	467	GAC(D)	GAT(D)	Synonymous	Neutral
L	528	AAG(K)	AAA(K)	Synonymous	Neutral
L	554	ATT(I)	ATC(I)	Synonymous	Purifying
L	570	TTG(L)	CTG(L)	Synonymous	Neutral
L	606	TAT(Y)	TAC(Y)	Synonymous	Purifying
L	653	CCA(P)	CCG(P)	Synonymous	Purifying
L	689	AGT(S)	AGC(S)	Synonymous	Purifying
L	717	GTT(V)	GTC(V)	Synonymous	Neutral
L	762	GTA(V)	ATA(I)	Nonsynonymous	Neutral
L	847	TTG(L)	CTG(L)	Synonymous	Neutral
L	871	TCT(S)	TCC(S)	Synonymous	Purifying
L	916	TTA(L)	GTA(V)	Nonsynonymous	Neutral
L	958	GGG(G)	GGA(G)	Synonymous	Purifying
L	968	GAG(E)	GAA(E)	Synonymous	Neutral
L	1069	CTT(L)	CTC(L)	Synonymous	Purifying
L	1074	GGG(G)	GGA(G)	Synonymous	Purifying
L	1124	AAC(N)	AAT(N)	Synonymous	Purifying
L	1126	TCC(S)	TCA(S)	Synonymous	Purifying
L	1158	TCC(S)	TCT(S)	Synonymous	Purifying
L	1190	GTT(V)	ATT(I)	Nonsynonymous	Neutral
L	1222	ACA(T)	ACG(T)	Synonymous	Purifying
L	1237	GGC(G)	GGT(G)	Synonymous	Purifying
L	1242	CAG(Q)	CAA(Q)	Synonymous	Purifying
L	1258	AGG(R)	AGA(R)	Synonymous	Neutral
L	1268	TTG(L)	TTA(L)	Synonymous	Purifying
L	1452	CTG(L)	CTT(L)	Synonymous	Purifying
L	1461	GAA(E)	GAG(E)	Synonymous	Purifying
L	1513	GTT(V)	GTC(V)	Synonymous	Purifying
L	1519	CTC(L)	CTT(L)	Synonymous	Purifying
L	1526	ACT(T)	ACC(T)	Synonymous	Neutral
L	1536	TCT(S)	TCC(S)	Synonymous	Neutral
L	1569	TTT(F)	TTC(F)	Synonymous	Neutral
L	1622	CCA(P)	CTA(L)	Nonsynonymous	Diversifying
L	1644	TTA(L)	TTG(L)	Synonymous	Neutral
L	1712	GCT(A)	GCC(A)	Synonymous	Purifying
L	1760	GTG(V)	GTC(V)	Synonymous	Purifying
L	1784	CGG(R)	CAG(Q)	Nonsynonymous	Diversifying
L	1824	ACA(T)	ACT(T)	Synonymous	Neutral
L	1856	AAT(N)	AAC(N)	Synonymous	Purifying
L	1876	AGG(R)	AAG(K)	Nonsynonymous	Neutral
L	1915	GCG(A)	TCG(S)	Nonsynonymous	Neutral
L	1930	ATT(I)	ATC(I)	Synonymous	Neutral
L	1960	ATT(I)	GTT(V)	Nonsynonymous	Neutral
L	2010	AAA(K)	AAG(K)	Synonymous	Neutral
L	2021	CCA(P)	CCG(P)	Synonymous	Purifying
L	2040	CTG(L)	TTG(L)	Synonymous	Purifying
L	2062	CGT(R)	CGC(R)	Synonymous	Purifying

Figure S1. Differential mutations at multiple gene codon regions between 98COE and IN0919WYB2. Information presented in figure S1 regarding the evolutionary relevance of the

differential mutations (impacting multiple codons) between the VSIV strains 98COE and IN0919WYB2, was obtained for a previous study [4]. The selection class of different codons was inferred by the method Mixed Effects Model of Evolution (MEME). The alignment used for this analysis included viral sequences representing the genetic diversity of VSIV in nature [4]. Sites were classified in three different selection classes. Diversifying selection: Natural selective force that promotes adaptation and innovation by increasing amino acid diversity in a viral protein. Purifying selection: Natural selective force that favors the amino acid conservation in a viral protein. It results in a stabilizing selection that preserves the functionality of a protein by removing deleterious alleles from the viral population. Neutral evolutions: Changes in the genome that are caused by random genetic drift, rather than natural selection.

A

Dilution	TCID50/mL	Corrected OD	Result	CT value
Undiluted	$1x10^{8.43}$	3.688	Positive	11.57
$1x10^{-1}$	$1x10^{7.43}$	3.831	Positive	14.41
$1x10^{-2}$	$1x10^{6.43}$	2.171	Positive	17.8
$1x10^{-3}$	$1x10^{5.43}$	0.252	Positive	21.29
$1x10^{-4}$	$1x10^{4.43}$	0.038	Negative	24.59
$1x10^{-5}$	$1x10^{3.43}$	0.021	Negative	27.92
$1x10^{-6}$	$1x10^{2.43}$	0.013	Negative	31.39
$1x10^{-7}$	$1x10^{1.43}$	0.016	Negative	34.93

B

Dilution	TCID50/mL	Corrected OD	Result	CT value
Undiluted	$1x10^{9}$	3.514	Positive	12.95
$1x10^{-1}$	$1x10^{8}$	3.562	Positive	15.91
$1x10^{-2}$	$1x10^{7}$	2.51	Positive	19.27
$1x10^{-3}$	$1x10^{6}$	0.321	Positive	22.66
$1x10^{-4}$	$1x10^{5}$	0.049	Negative	26.04
$1x10^{-5}$	$1x10^{4}$	0.034	Negative	29.3
$1x10^{-6}$	$1x10^{3}$	0.013	Negative	32.97
$1x10^{-7}$	$1x10^{2}$	0.02	Negative	36.56

C

sample ID	Source	Corrected OD	Result	Ct value
P302 2DPI VI-NS	Viral isolation	3.796	Positive	13.53
P309 4DPI VI-NS	Viral isolation	3.94	Positive	13.94
P304 4DPI VI-NS	Viral isolation	3.815	Positive	14.07
P310 4DPI VI-NS	Viral isolation	2.929	Positive	14.29
P307 4DPI VI-NS	Viral isolation	3.813	Positive	14.87
P310 6DPI VI-OS	Viral isolation	3.684	Positive	15.29
P300 4DPI VI-OS	Viral isolation	3.923	Positive	15.47
P301 2DPI VI-NS	Viral isolation	3.765	Positive	16.06
P302 6DPI VI-NS	Viral isolation	3.707	Positive	17.32
P302 6DPI NS	Clinical Sample/Nasal swab	0.616	Positive	20
*P302 7DPI NS	Clinical Sample/Nasal swab	0.033	Negative	22.75
*P307 6DPI NS	Clinical Sample/Nasal swab	0.121	Positive	22.97
*P310 8DPI OS	Clinical Sample/Oral swab	0.119	Positive	23.46
*P300 6DPI NS	Clinical Sample/Nasal swab	0.021	Negative	23.63
*P305 8DPI NS	Clinical Sample/Nasal swab	0.164	Positive	24.76
*P307 7DPI NS	Clinical Sample/Nasal swab	0.014	Negative	26.49
VSIV/Positive control	Viral stock	1.573	Positive	NA

IN0919WYB1 **98COE**

Figure S2. Evaluation of clinical samples found positive by RT-qPCR using DAS ELISA. Information presented in figure S2. A) Multiple tenfold dilutions of the viral stock IN0919WYB2 (titer $1 \times 10^{8.43}$ $TCID_{50}$/mL) were conducted to compare the limit of detection between qRT-PCR (results expressed as CT values) and DAS ELISA (results expressed as corrected OD, cutoff >0.1). B) Multiple tenfold dilutions of the viral stock 98COE (titer 1×10^{9} $TCID_{50}$/mL) were conducted to compare the limit of detection between qRT-PCR (results expressed as CT values) and DAS ELISA (results expressed as correct-ed OD, cutoff >0.1). C) Multiple samples from the animal experiments were evaluated by DAS ELISA and qRT-PCR, it included: viral isolates and clinical samples positive by qRT-PCR but negative for viral isolation in Vero cells (samples with asterisks). Clinical sample P302 6DPI NS was used as a control to assess if a clinical sample positive for viral isolation and qRT-PCR was also positive by DAS ELISA.
